# Analysis of VOCs Emitted from Small Laundry Facilities: Contributions to Ozone and Secondary Aerosol Formation and Human Risk Assessment

**DOI:** 10.3390/ijerph192215130

**Published:** 2022-11-16

**Authors:** Da-Mee Eun, Yun-Sung Han, Soo-Hyun Park, Hwa-Seong Yoo, Yen Thi-Hoang Le, Sangmin Jeong, Ki-Joon Jeon, Jong-Sang Youn

**Affiliations:** 1 Department of Energy and Environmental Engineering, The Catholic University of Korea, Bucheon 14662, Republic of Korea; 2 Lab.SolEmis, Incheon 22212, Republic of Korea; 3 Department of Environmental Engineering, Inha University, Incheon 22212, Republic of Korea; 4 Program on Environmental and Polymer Engineering, Inha University, Incheon 22212, Republic of Korea; 5 Department of Chemistry, University of Massachusetts, Lowell, MA 01854, USA; 6 Particle Pollution Research and Management Center, Incheon 21999, Republic of Korea

**Keywords:** volatile organic compound, risk assessment, laundry, indoor air quality, secondary organic aerosol

## Abstract

Volatile organic compounds (VOCs) emitted to the atmosphere form ozone and secondary organic aerosols (SOA) by photochemical reactions. As they contain numerous harmful compounds such as carcinogens, it is necessary to analyze them from a health perspective. Given the petroleum-based organic solvents used during the drying process, large amounts of VOCs are emitted from small laundry facilities. In this study, a laundry facility located in a residential area was selected, while VOCs data emitted during the drying process were collected and analyzed using a thermal desorption-gas chromatography/mass spectrometer (TD-GC/MS). We compared the results of the solvent composition, human risk assessment, contribution of photochemical ozone creation potential (POCP), and secondary organic aerosol formation potential (SOAP) to evaluate the chemical species. Alkane-based compounds; the main components of petroleum organic solvents, were dominant. The differences in evaporation with respect to the boiling point were also discerned. The POCP contribution exhibited the same trend as the emission concentration ratios for nonane (41%), decane (34%), and undecane (14%). However, the SOAP contribution accounted for o-xylene (28%), decane (27%), undecane (25%), and nonane (9%), thus confirming the high contribution of o-xylene to SOA formation. The risk assessment showed that acrylonitrile, carbon tetrachloride, nitrobenzene, bromodichloromethane, and chloromethane among carcinogenic compounds, and bromomethane, chlorobenzene, o-xylene, and hexachloro-1, 3-butadiene were found to be hazardous, thereby excessing the standard value. Overall these results facilitate the selection and control of highly reactive and harmful VOCs emitted from the dry-cleaning process.

## 1. Introduction

Volatile organic compounds (VOCs) are carbon-based chemicals that can easily evaporate into the atmosphere due to their high vapor pressure [1]. VOCs are originated from natural or anthropogenic sources. The anthropogenic sources include motor vehicle exhausts, combustion processes utilizing fossil fuels, energy storage and distribution, petroleum solvent usage, and other industrial processes [2]. VOCs have the characteristic that fugitive emissions are large and important among these anthropogenic sources [3]. Therefore, research to observe and analyze specific emission sources is particularly important, and many related studies have been conducted [4,5,6,7]. Of them, the use of organic solvents accounted for >50% of Korea’s total VOC emissions in 2019 (CAPSS). In domestic small laundry facilities, various VOC emissions from petroleum-based organic solvents cause serious indoor pollution [8]. Consequently, several studies have focused on the risk of VOCs emitted during the dry-cleaning process [8,9,10,11,12,13,14,15].

When VOCs are released into the atmosphere, ozone, and secondary organic aerosols (SOA) are formed by photochemical reactions [13,16]. Ozone is produced by the atmospheric oxidation of VOCs with nitrogen oxides when sunlight is present [17,18,19,20]. Likewise, secondary organic aerosol formation occurs by the oxidation of VOCs, thereby becoming a major factor behind fine PM_2_._5_ pollution [21,22,23,24]. Lee et al., (2021) [25] investigated VOC emissions during the dry-cleaning process, while also determining decane and undecane as two chemical species with the strongest influence on ozone and SOA creation.

As indoor life accounted for the majority of daily routine during COVID-19 pandemic, indoor pollution has received considerable attention in research [26,27]. Numerous previous studies have shown that indoor pollutant concentrations are more dominant than those outdoors [28,29,30]. It has been estimated that 4.3 million people worldwide die prematurely from diseases caused by indoor air pollution every year [31]. Inhalation is the main route of VOC exposure to the human body [32]. In small laundry facilities, chemicals with carcinogenic or other hazards can pose a significant threat to the workers and surrounding residents, thereby putting them under a high risk of exposure. According to Çankaya et al., (2018) [10], dry cleaners exhibited the second highest hazard quotient among the handicraft workplaces (restaurants, dry cleaners, photocopy centers, and auto paint shops), whereas bromoform exhibited the highest carcinogenic risk (1.36 × 10^−5^) in dry cleaners. Niu et al., (2021) [14] considered the chemical species of VOCs emitted from 13 various volatile emission sources, and analyzed the temperature dependence of source profiles. To this end, they compared how different VOC mass fractions in summer and winter were formed at 5 workplaces, including laundry. They found that halohydrocarbons were dominant in winter while alkanes prevailed in summer.

However, these studies were limited because they analyzed ozone and SOA formation and human risk for VOCs emitted during dry-cleaning process or were focused on the concentration of indoor pollution concentration according to the temperature change. In particular, the studies on risk assessment are extremely scantly over the past 15 years, especially those specifically focused only on domestic laundry facilities. Independent national-scale studies are needed because atmospheric environments, the dry-cleaning solvents mainly used, and the composition of the workplace; all differ depending on the country. In this study, a comprehensive assessment was conducted alongside the analysis of the contribution to ozone and SOA formation. These results can be used as the data in future for independent studies focused on Korea. The VOCs emitted during dry-cleaning process in small laundry facilities were analyzed using thermal desorption-gas chromatography/mass spectrometer (TD-GC/MS). Moreover, (1) the contribution to ozone creation was determined according to the analysis results using photochemical ozone creation potential (POCP) value; (2) the contribution to SOA generation using secondary organic aerosols potential (SOAP) value was determined as well, (3) while deterministic risk assessment was performed to reflect each VOCs species.

## 2. Materials and Methods

### 2.1. VOCs Sampling and Analysis

To date, domestic dry cleaners and small household laundry account for 98% of the overall laundry industry [33]. Petroleum solvents and non-petroleum solvents such as perchloroethylene and fluorine-based solvents are used as laundry solvents. It is known that >95% of domestic laundry facilities use petroleum-based solvents (hydrocarbons) [34]. Petroleum distillates are composed of alkanes, cycloalkanes, and aromatic compounds [13]. Moreover, petroleum laundry solvents used in the dry-cleaning process are discharged into the atmosphere during the washing process, solvent circulation filtration process, and drying process [34]. In this study, a dry washing machine was used in a small laundry facility. Methodologically, 1 kg of cotton fiber was washed using a petroleum laundry solvent. The dry-cleaning process lasted for 23 min: (1) main washing (10 min), (2) rinse washing (5 min), (3) deoiling process (8 min).

To analyze the samples emitted from the dry-cleaning process, gas chromatography-mass spectrometer (GC-MS) was used. For the reproducibility of the results, the same experiment was performed 3 times. The gas was collected inside the laundry with flow adjustable mini pump (Amos 100, YTK Co., Uiwang, Korea) and a solid sorbent tube (3.5” x 1/4”, Markes, Llantrisant, UK) filled with triple adsorption trap (Carbopack C, Carbopack B, Carbosieve SIII). The sorbent tube demonstrated excellent adsorption and desorption capabilities for VOCs in a wide range from C2 to C20 among the adsorbents, thereby implying that it can be efficiently collected (details are summarized in Table S1). The samples collected in adsorption tubes were stored in a desiccator to maintain temperature and humidity. The analysis was conducted as quickly as possible.

**Figure 1:**
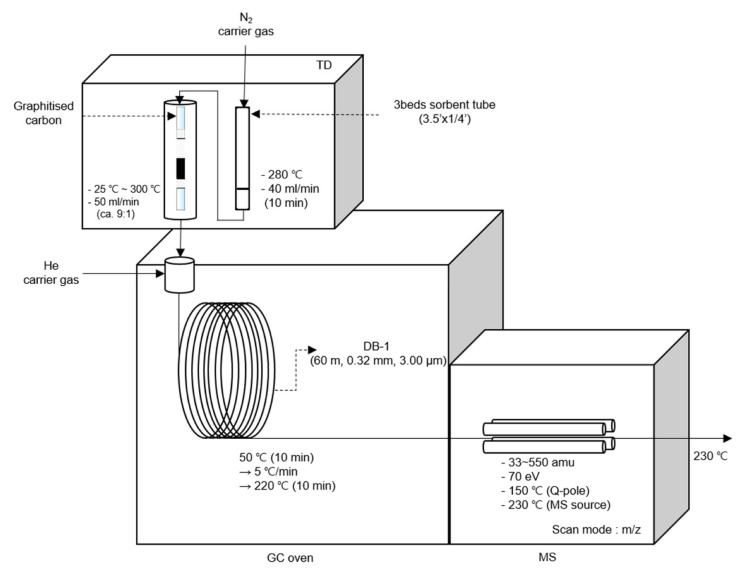
Operating conditions of thermal desorption and GC/MS used for VOCs analysis.

The collected sample was quantitatively and qualitatively analyzed using a thermal desorption gas chromatography/mass spectrometer (GCMS: 5977B, Agilent Technologies, Santa Clara, CA, USA). In the thermal desorption (TD: TD100xr, Markes, Llantrisant, UK), the adsorbed sample was thermally desorbed (280 °C, 40 mL/min, 10 min). The primary desorbed sample was moved to the cold trap through N_2_ gas (99.999%), and for secondary desorption, the temperature was increased to 300 °C. The initial temperature of the GC oven was 50 °C, held for 10 min and subsequently increased up to 220 °C, and then, held for another 10 min. After passing He (99.999%) through the column at the flow rate of 2.5 mL/min, it was analyzed by a quadrupole mass spectrometer. The sample was qualitatively analyzed using the scan mode. The detailed operating conditions are shown in Figure 1. A total of 77 species were used for qualitative and quantitative analysis (Table S2).

### 2.2. Estimation of POCP and SOAP 

Ground level ozone is a secondary pollutant produced by the reaction of volatile organic compounds (VOCs) and nitrogen oxides (NOx) in the presence of sunlight in the atmosphere [35]. VOCs species differ in their reactivity and structure, thus implying that the extent to which they contribute to ozone formation varies from species to species. Thus, the emissions and photochemical reactivity should be both estimated [36].

The method, proposed by Derwent et al., (2007) [37] reflects the contribution to photochemical ozone creation (POC) in a semi-quantitative way by considering the emission and chemical reactivity of each VOCs. However, as the POCP value was determined by a realistic air mass trajectory model across northeast Europe, it could emerge in different ways depending on the weather and spatial characteristics of Korea [25]. The POCP value was expressed by calculating the ozone increment of a particular hydrocarbon based on the ozone increment of ethylene (POCP = 100). Equations (1) and (2) are applied for POCP calculation.

(1) $$POCP_{i}=\frac{Ozone\;increment\;with\;the\;ith\;hydrocarbon}{Ozone\;increment\;with\;ethylene}\times 100$$

The contribution of ozone creation by VOCs was calculated using the *POCP_i_* suggested by Derwent et al., (2007) [37].

(2) $$POCP=C_{i}\times POCP_{i}$$

*C_i_* is the concentration of VOCs emitted per dry-cleaning cycle, and *POCP* is the contribution value of ozone creation expressed by multiplying the emission by the *POCP_i_*.

Secondary organic aerosols (*SOA*), which account for a significant fraction of PM_2_._5_ in the atmosphere, are formed through the oxidation of VOCs precursors [13]. Equations (3) and (4) are applied for SOAP calculation.

(3) $$SOAP_{i}=\frac{Increment\;in\;SOA\;mass\;concentration\;with\;species,\;i}{Increment\;in\;SOA\;with\;toluene}\times 100$$

The *SOAP_i_* reflects the extent to which the compound forms an *SOA* when an additional mass concentration is present, compared to the SOA formed when the same amount of toluene is present [38]. SOAPs were expressed as an index relative to toluene = 100.

(4) $$SOAP=C_{i}\times SOAP_{i}$$

*SOAP* is the contribution value of secondary organic aerosols formed expressed by multiplying the emission by the *SOAP_i_*.

### 2.3. Risk Assessment

Risk assessment is the scientific process for characterizing the nature and magnitude of risks to human health, thereby elucidating the mechanisms of events behind the adverse health effects, and deepening the knowledge about epidemiology [39,40]. Each factor required for the exposure assessment can be calculated in a deterministic or probabilistic manner depending on the purpose [41]. Since the deterministic model uses a single value for the model parameters, it can advantageously estimate typical exposure differently from the values of the probabilistic model, thus allowing an easy interpretation of results [39]. In the United States, the product inventory and related toxicity data have been previously reviewed by DeLeo et al., (2018) [42] within the Cleaning Product Ingredient Safety Initiative (CPISI). However, the deterministic risk assessment is still ambiguous for cleaning product ingredients of all the products, including laundry care products, and safety data; established for some products.

In this study, to elucidate the effects on the human body of inhaling VOCs emitted during the dry-cleaning process, a human risk assessment was evaluated for VOCs with toxic data. The applied procedures for risk assessment designed by the National Research Council (NRC) included hazard identification, dose response assessment, exposure assessment, and risk characterization. Carcinogenic and non-carcinogenic compounds were analyzed using hazard identification and dose–response assessment. For carcinogenic and non-carcinogenic substances, 11 and 8 VOCs were selected from the International Agency for Research on Cancer (IARC) and the Integrated Risk Information System (IRIS), respectively. Inhalation slope factor (mg/kg·day)^−1^ for carcinogenic substances and the reference concentration (mg/m^3^) for non-carcinogenic substances were obtained. The inhalation slope coefficient generally represents the cancer risk per unit dose of a pollutant and is defined as the value close to the 95% confidence level for an increased risk of carcinogenesis through lifetime inhalation exposure [43]. 8 carcinogenic compounds ranged from 3.50 × 10^−5^ (methylene chloride) to 2.38 × 10^−1^ (acrylonitrile); benzene was a class 1 carcinogen, methylene chloride was class 2A, and acrylonitrile, carbon tetrachloride, nitrobenzene, bromodichloromethane, and 4-methyl-2-pentanone were designated as the class 2 B carcinogens by the IARC (Table 1). The reference concentration reflects an estimate of a human continuous inhalation exposure [44], and the values for the 11 non-carcinogens ranged from 3.50 × 10^−3^ (hexachloro-1,3-Butadiene) to 2.17 × 10^2^ (ethanol) (Table 2).

Moreover, the dose-response data of carcinogenic and non-carcinogenic compounds such as tumor type and test species of each substance were obtained (Tables S3 and S4). If the evaluation is performed using animal experimental data due to a lack of data, the interpretation of the analyzed data was scrupulous. The results could emerge as errors in the process of converting the capacity to apply to the human body [45].

The exposure assessment is performed through the estimation of the exposure level at which the chemicals enter the human body according to the analysis data of chemicals in the environment [45]. Life average daily dose (*LADDs*; mg/kg·day) can be calculated according to Equation (5).

(5) $$LADDs=\frac{CA\times IR\times ED\times EF\times L}{BW\times ATL\times NY}$$

*CA* is the contaminant concentration (mg/m^3^); *IR* is the inhalation rate (m^3^/h); *ED* is the exposure duration (h/week); *EF* is the exposure frequency (week/yr); *L* is the length of exposure (yr); *BW* is the body weight (kg); *ATL* is the average time of lifetime (70 years); *NY* is the number of days per year (365 days/year). For carcinogenic substances, *LADDs* and an inhalation slope factor both could be used to quantify the cancer risk, while for non-carcinogenic substances, the hazard quotient was determined using the reference concentration. An acceptable limit was suggested at the risk level (1.00 × 10^−6^), at which in one in a million, cancer would be developed (e.g., the naturally occurring probability of cancer). Furthermore, the comparison between LADDs for the current atmospheric level with the reference value was performed, thereby reflecting that the adverse effects of non-carcinogens are likely to occur if the hazard quotient exceeds 1 [46].

## 3. Results and Discussion

### 3.1. Concentrations of VOCs during Dry Cleaning Process

Of the 77 analytes, 47 were detected during the dry-cleaning process, which accounted for ~61% of the total. The quantitative results were calibrated using the laundry indoor temperature and pressure (20 °C, 1 atm). The five substances emitted the most were determined: nonane (409.2 ppb), decane (319.9 ppb), undecane (127.4 ppb), nonanal (54.2 ppb), and decanal (29.1 ppb), which accounted for about 85% of the total emission concentration (Table S5).

**Figure 2:**
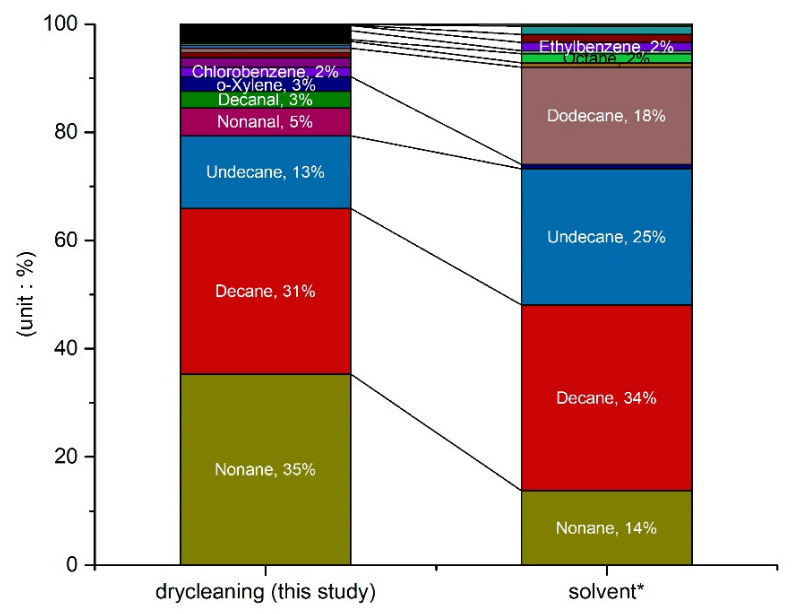
VOCs composition of organic solvents and dry-cleaning emissions. * Ref [47].

For the dry-cleaning process, a petroleum-based organic solvent was used. Figure 2 shows the comparison of the organic solvent release test [47] with the results of this study (in %). The emission concentrations of the alkane-based species from the dry-cleaning process were determined as well: nonane (35%), decane (31%), undecane (13%), and dodecane (1%); all accounted for 82% of the total emission concentration. In the composition of the organic solvent, decane (34%), undecane (25%), dodecane (18%), and nonane (14%) were confirmed to be dominant at 94% of the C9–C12 alkane based chemical species, as shown in Figure 2. As seen, the VOCs emitted during the dry-cleaning process were strongly affected by the organic solvent used. A previous study indicated that the solvent composed of perchloroethylene (70%) and naphtha gas (30%) in Mexico, while halohydrocarbons accounted for 96.2% of the total emission mass [48], and 100% in Chicago [49]. In line with the findings from Lee et al., (2021) [25], we found that besides the type of organic solvent used, the discharged concentration varied according to different operational conditions like operating temperature, operating time, type of laundry, and weight of laundry.

Moreover, the comparison of dry-cleaning emissions with the organic solvent demonstrated that nonane seemingly increased from 14% to 35%, while decane and undecane seemingly decreased from 34% to 31% and from 25% to 13%. The compounds with low boiling points are generally characterized by the ability to evaporate into the atmosphere, mixing with air, and accumulation ability [50]. Besides that, Wang et al., (2013) [51] have previously confirmed that the evaporation rate of the solvent varied according to the difference in its boiling point. In the nonane solvent, having a relatively higher boiling point than the isooctane solvent, the evaporation was more strongly inhibited and the liquid state was maintained. In the dry cleaning process of this study, where the internal temperature of the device was maintained at 140–160 °C, the evaporation of nonane (B.P: 151 °C) in the total evaporation would be higher than that for undecane (B.P: 196 °C) and dodecane (B.P: 216 °C).

### 3.2. Photochemical Ozone Creation Potential (POCP) and Secondary Organic Aerosol Formation Potential (SOAP) of VOCs

**Figure 3:**
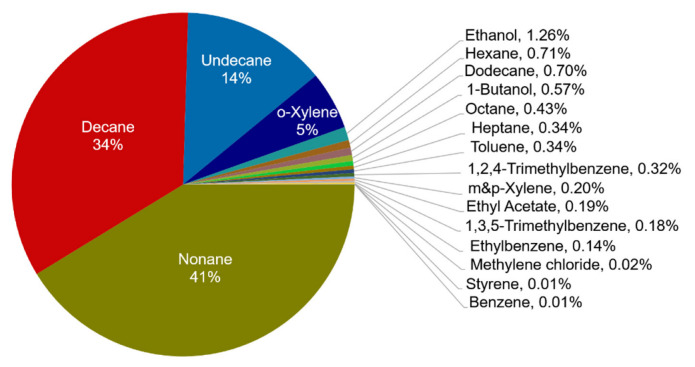
POCP contributions (%) of VOCs emitted from one cycle of dry-cleaning process.

VOCs emitted from small-scale indoor emission sources infiltrate to ambient air and have a great effect on ozone and SOA formation by photochemical reactions [52]. Among the substances emitted from the dry-cleaning process, 19 chemical species to which the POCP value can be applied were identified; they accounted for 40%. The total contribution of 19 substances was found to be 33.7 ppm, and nonane (41.3%), decane (34.2%), undecane (13.6%), and o-xylene (5.5%) accounted for ~95% of the contribution rate of ozone creation (Figure 3). As nonane, decane, and undecane accounted for ~80% of the VOCs emitted from the dry-cleaning process, the contribution rate to the ozone creation was estimated to be high separately from the POCP value. Furthermore, o-xylene, which accounted for 3% of the total emission concentration contributed 5.5% to the ozone creation. Lee et al. (2021) [25] have previously reported that decane (32.4%), nonane (21.7%), undecane (18.3%), Hexane (15.5%), and octane (4.6%) exhibited the highest contribution to the ozone formation. Lee et al., (2021) [25] found the same top three compounds but their contribution rates differed. Moreover, types and contribution rates of the remaining compounds were also different. This difference was in turn driven by the difference in the chemical compositions of various organic solvents, dry-cleaning equipment, weight, and type of laundry [25].

**Figure 4:**
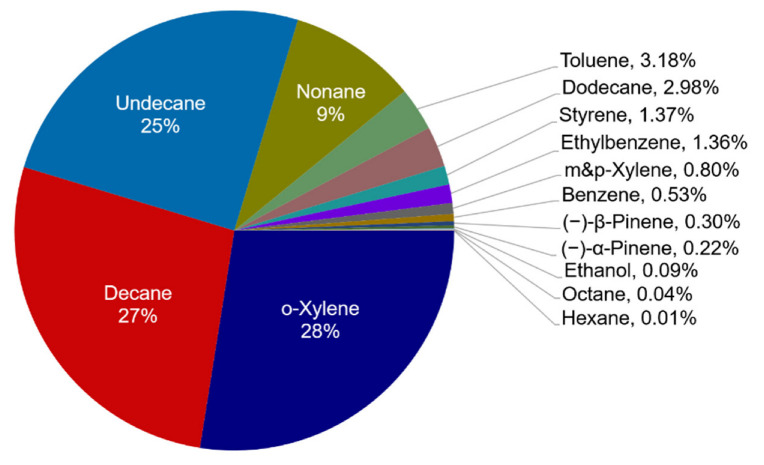
SOAP contributions (%) of VOCs emitted from one cycle of dry-cleaning process.

Of the substances emitted from the dry-cleaning process, 18 chemical species to which SOAP value can be applied, were identified; they accounted for 38% of the total. The total SOAP contribution of the substances was found to be 8.3 ppm. Unlike POCP, o-xylene (27.5%) exhibited the highest contribution rate, while decane (27.2%) and undecane (25.0%) exhibited similar but lower contribution rates. Next, nonane (9.4%) and toluene (3.2%) exhibited high contribution, and the contribution rate of the five VOCs to SOA formation accounted for ~92% of the total (Figure 4). O-xylene accounted for 3% of the total dry-cleaning emissions. However, the driver behind their high contribution to SOA formation was related to stronger exposure to the SOAP value of the compounds, compared with the emission concentration. This indicates that their VOCs significantly affected SOA formation. Lee et al., (2021) [25] have previously shown that undecane (44.8%), decane (30.4%), dodecane (9.3%), toluene (5.5%), and nonane (5.5%) exhibited the highest contribution to SOA formation. In turn, we revealed some differences because o-xylene was not detected in previous study.

### 3.3. Risk Assessment

**Figure 5:**
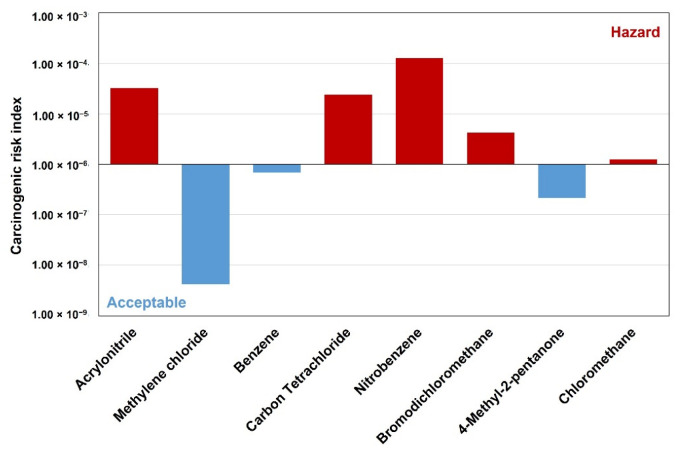
Risk assessment result of toxic carcinogenic VOCs.

Figure 5 shows the evaluation for the selected 8 carcinogens. As seen, the mean total estimated cancer risk was 2.36 × 10^−5^. Of the 8 VOCs, nitrobenzene is the substance with the highest cancer risk. Chemical species are considered to be hazardous because their cancer risks exceed the carcinogenic standard (1.00 × 10^−6^) are acrylonitrile (3.26 × 10^−5^), carbon tetrachloride (2.40 × 10^−5^), nitrobenzene (1.26 × 10^−4^), bromodichloromethane (4.19 × 10^−6^), and chloromethane (1.25 × 10^−6^), which are classified as 4 types of 2B class carcinogens and 1 type of class 3 carcinogen. Thus, they were seemingly directly exposed to the risk of kidney cancer, liver cancer, respiratory cancer, etc. Moreover, for benzene, a class 1 carcinogen, it was below the standard value, but close to the standard value (6.84 × 10^−7^). Thus, it is necessary to pay attention to it.

**Figure 6:**
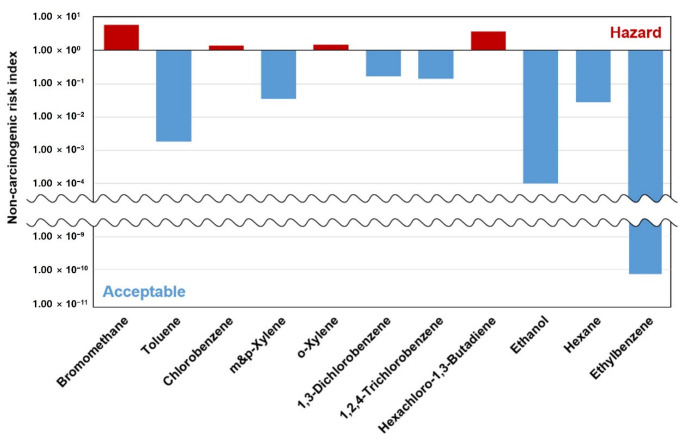
Risk assessment result of toxic non-carcinogenic VOCs.

Figure 6 shows the evaluation of 11 non-carcinogenic substances. As seen, the mean total hazard quotient was 1.19. Of the 11 compounds, the non-carcinogenic risk index of bromomethane was 5.95, thereby manifesting the highest value. Some compounds were deemed to be hazardous because the emission concentration exceeded the standard concentration for bromomethane (5.95), chlorobenzene (1.45), o-xylene (1.53), and hexachloro-1,3-Butadiene (3.84), 4 out of 11. Moreover, the hazard quotient of 1,3-dichlorobenzene (0.17) and 1,2,4-trichlorobenzene (0.14) was below the standard value, but close to the standard value, thus implying that such a result cannot be neglected. Through the inhalation of these substances, laundry workers and people living in the vicinity are likely to suffer from toxic effects on the nervous, respiratory, urinary, and hepatic systems through inhalation. In particular, small-scale laundries are concentrated in residential areas, thus posing a major and significant health risk to the surrounding population. However, the emission concentrations were estimated using a single measurement and the number of data resulted in large standard deviations, thereby manifesting a considerable limitation [46]. Çankaya et al., (2018) [10] have previously indicated that carbon tetrachloride (7.65 × 10^−6^), benzene (1.09 × 10^−5^), and bromodichloromethane (4.53 × 10^−6^); all pose cancer risks. Although carbon tetrachloride exhibited a higher risk in this study, benzene and bromodichloromethane had been shown to have a slightly higher level of risk in the previous study. Moreover, even though chlorobenzene and o-xylene were found to be harmful in this study, toluene (2.12 × 10^−2^), chlorobenzene (4.05 × 10^−2^), ethylbenzene (5.06 × 10^−4^), and xylenes (1.59 × 10^−2^); all did not exceed the standard, thereby signifying acceptable levels [10]. Omrane, F., et al., (2021) [53] previously found that the carcinogenic risks of TCE and PCE in Tunisia using chlorinated solvents were 1.65 × 10^−1^ and 1.18 × 10^−2^, respectively, thus greatly exceeding the standard. It is therefore reasonable to suggest that they pose a high possibility of causing liver or kidney cancer.

## 4. Conclusions

In this study, VOCs emitted from the dry-cleaning process of small laundry facilities were analyzed, while the contribution of the chemicals to ozone and SOA formation was quantified. Furthermore, the risk of toxic substances to the human body was assessed and discussed for each chemical. We used the samples taken from dry-cleaning processes in small laundry facilities and detected 47 of 77 analytes. We identified the top three substances of dry-cleaning emissions as follows: nonane (35%), decane (31%), and undecane (13%). The dominant proportion of the alkane (82%) was affected by the organic solvent, mostly composed of alkane (96%). Moreover, we concluded that the volatilization amount of nonane was higher than that of other compounds under the influence of the internal temperature of the dry-cleaning process. The analysis of POCP and SOAP contribution confirmed that the contribution to ozone formation was dominated by nonane (41%), decane (34%), and undecane (14%) with high emission concentrations. We also analyzed the SOA formation contribution and found that o-xylene exhibited the highest contribution rate (28%) because the SOAP value had a greater effect, compared with that of the emission concentration. Thus, we confirmed that aromatic compounds greatly contributed to the formation of SOA. Furthermore, decane (27%) and undecane (25%) exhibited low but similar levels. The health risk assessment demonstrated that (1) acrylonitrile, carbon tetrachloride, nitrobenzene, bromodichloromethane, and chloromethane exceeded the standard for carcinogenic substances; (2) benzene, class 1 carcinogen was close to the standard; (3) bromomethane, chlorobenzene, o-xylene, and hexachloro-1,3-butadiene exceeded the standard for non-carcinogenic substances (all these results were statistically significant). Thus, people working in small laundry facilities are exposed to health risks. It is also plausible that the period of exposure to toxic substances is high because they reside indoors for a long time.

It is necessary to remove specific chemical substances from the organic solvent itself or to limit the emission by installing a reduction device at the outlet of dry-cleaning machine. Moreover, laundry workers should be generally aware of the potential health risks they are exposed to and must wear personal protective equipment. After the dry-cleaning process, it is necessary to minimize the risk to the human body by providing sufficient ventilation of the indoor air. These findings lay the foundation for future studies, aimed at preparing VOC regulatory standards and management plans.

## Figures and Tables

**Table 1:** Dose–response data of toxic carcinogenic VOCs.

No.	Compound	CAS NO.	IARC WOE *	EPA WOE **	Unit Risk (µg/m^3^)^−1^	Inhalation Slope Factor (mg/kg·day)^−1^	Reference
1	Acrylonitrile	107-13-1	2B	B1	6.80 × 10^−5^	2.38 × 10^−1^	EPA IRIS
2	Methylene chloride	75-09-2	2A	LH	1.00 × 10^−8^	3.50 × 10^−5^	EPA IRIS
3	Benzene	71-43-2	1	CH	7.80 × 10^−6^	2.73 × 10^−2^	EPA IRIS
4	Carbon Tetrachloride	56-23-5	2B	LH	1.50 × 10^−5^	1.30 × 10^−1^	EPA IRIS
5	Nitrobenzene	98-95-3	2B	LH	4.00 × 10^−5^	1.40 × 10^−1^	EPA IRIS
6	Bromodichloromethane	75-27-4	2B	-	1.77 × 10^−5^	6.20 × 10^−2^	MDEQ
7	4-Methyl-2-pentanone	108-10-1	2B	lnl	4.94 × 10^−7^	1.73 × 10^−3^	MDEQ
8	Chloromethane	74-87-3	3	lnl	1.80 × 10^−6^	6.30 × 10^−3^	HEAST, MDEQ

* IARC WOE (weight of evidence for carcinogenicity): 1—carcinogenic; 2A—probably carcinogenic; 2B—possibly carcinogenic; 3—not classifiable; 4—probably not carcinogenic). ** EPA WOE: A—human carcinogen; B1—probable carcinogen, limited human evidence; B2—probable carcinogen, sufficient evidence in animals; C—possible human carcinogen; D—not classifiable; E—evidence of noncarcinogenicity; CH—carcinogenic to humans; LH—likely to be carcinogenic; SE—suggestive evidence of carcinogenic potential; InI—inadequate information to assess carcinogenic potential; NH—not likely to be carcinogenic).

**Table 2:** Dose–response data of toxic non-carcinogenic VOCs.

No.	Compound	CAS NO.	RfD * (mg/kg·day)	RfC ** (mg/m^3^)	Reference
1	Bromomethane	74-83-9	1.40 × 10^−3^	5.00 × 10^−3^	EPA IRIS
2	Toluene	108-88-3	8.00 × 10^−2^	5.00 × 10^0^	EPA IRIS
3	Chlorobenzene	108-90-7	2.00 × 10^−2^	7.00 × 10^−2^	EPA IRIS
4	m&p-Xylene	1330-20-7	2.00 × 10^−1^	1.00 × 10^−1^	EPA IRIS
5	o-xylene	1330-20-7	2.00 × 10^−1^	1.00 × 10^−1^	EPA IRIS
6	1,3-Dichlorobenzene	541-73-1	2.00 × 10^−3^	7.00 × 10^−3^	ATSDR, MDEQ
7	1,2,4-Trichlorobenzene	120-82-1	1.00 × 10^−2^	3.50 × 10^−2^	EPA IRIS
8	Hexachloro-1,3-Butadiene	87-68-3	1.00 × 10^−3^	3.50 × 10^−3^	PPRTV
9	Ethanol	64-17-5	6.20 × 10^1^	2.17 × 10^2^	MDEQ
10	Hexane	110-54-3	-	7.00 × 10^−1^	EPA IRIS
11	Ethylbenzene	100-41-4	1.00 × 10^−1^	1.00 × 10^1^	EPA IRIS

* RfD: Reference dose, ** RfC: Reference concentration.

## Data Availability

Not applicable.

## Supplementary Materials

The following supporting information can be downloaded at: https://www.mdpi.com/article/10.3390/ijerph192215130/s1, Table S1: Characteristics of adsorbent used for VOCs sampling, Table S2: Chemical characteristics and method detection limit (MDL) of the analytes, Table S3: Information and health impact of toxic carcinogen VOCs, Table S4: Information and health impact of toxic non-carcinogen VOCs, Table S5: Concentration of VOCs emitted from one cycle of dry cleaning process.

## References

[1] Kamal M.S., Razzak S.A., Hossain M.M. (2016). Catalytic oxidation of volatile organic compounds (VOCs)—A review. Atmos. Environ..

[2] Cetin E., Odabasi M., Seyfioglu R. (2003). Ambient volatile organic compound (VOC) concentrations around a petrochemical complex and a petroleum refinery. Sci. Total Environ..

[3] Onat A. (2006). A review of fugitive emissions. Seal. Technol..

[4] Lin Q., Gao Z., Zhu W., Chen J., An T. (2023). Underestimated contribution of fugitive emission to VOCs in pharmaceutical industry based on pollution characteristics, odorous activity and health risk assessment. J. Environ. Sci..

[5] Sarkar C., Sinha V., Kumar V., Rupakheti M., Panday A., Mahata K.S., Rupakheti D., Kathayat B., Lawrence M.G. (2016). Overview of VOC emissions and chemistry from PTR-TOF-MS measurements during the SusKat-ABC campaign: High acetaldehyde, isoprene and isocyanic acid in wintertime air of the Kathmandu Valley. Atmos. Chem. Phys..

[6] Sarkar C., Sinha V., Sinha B., Panday A.K., Rupakheti M., Lawrence M.G. (2017). Source apportionment of NMVOCs in the Kathmandu Valley during the SusKat-ABC international field campaign using positive matrix factorization. Atmos. Chem. Phys..

[7] Stockwell C.E., Veres P.R., Williams J., Yokelson R.J. (2015). Characterization of biomass burning emissions from cooking fires, peat, crop residue, and other fuels with high-resolution proton-transfer-reaction time-of-flight mass spectrometry. Atmos. Chem. Phys..

[8] Song M., Kim K., Cho C., Kim D. (2021). Reduction of volatile organic compounds (Vocs) emissions from laundry dry-cleaning by an integrated treatment process of condensation and adsorption. Processes.

[9] Calvert G.M., Ruder A.M., Petersen M.R. (2011). Mortality and end-stage renal disease incidence among dry cleaning workers. Occup. Environ. Med..

[10] Çankaya S., Pekey H., Pekey B., Özerkan Aydın B. (2020). Volatile organic compound concentrations and their health risks in various workplace microenvironments. Hum. Ecol. Risk Assess..

[11] Ceballos D.M., Fellows K.M., Evans A.E., Janulewicz P.A., Lee E.G., Whittaker S.G. (2021). Perchloroethylene and Dry Cleaning: It’s Time to Move the Industry to Safer Alternatives. Front. Public Health.

[12] Kim J.H., Yoo K.S. (2017). Study on the enhancement of VOCs management at laundry facilities in Korea. J. Environ. Policy Adm..

[13] McDonald B.C., De Gouw J.A., Gilman J.B., Jathar S.H., Akherati A., Cappa C.D., Jimenez J.L., Lee-Taylor J., Hayes P.L., McKeen S.A. (2018). Volatile chemical products emerging as largest petrochemical source of urban organic emissions. Science.

[14] Niu Z., Kong S., Zheng H., Yan Q., Liu J., Feng Y., Wu J., Zheng S., Zeng X., Yao L. (2021). Temperature dependence of source profiles for volatile organic compounds from typical volatile emission sources. Sci. Total Environ..

[15] Park O.-H., Lee K.-S., Min K.-W., Cho G., Yoon K.-J., Jeong W.-S., Cho Y.-G., Kim E.-S., Yang J.-S. (2016). Generating characteristics of VOCs in a commercial laundry shop and the effects on the health of workers. J. Korean Soc. Occup. Environ. Hyg..

[16] Liu H., Liu S., Xue B., Lv Z., Meng Z., Yang X., Xue T., Yu Q., He K. (2018). Ground-level ozone pollution and its health impacts in China. Atmos. Environ..

[17] Lu X., Hong J., Zhang L., Cooper O.R., Schultz M.G., Xu X., Wang T., Gao M., Zhao Y., Zhang Y. (2018). Severe surface ozone pollution in China: A global perspective. Environ. Sci. Technol. Lett..

[18] Faust J.A., Wong J.P., Lee A.K., Abbatt J.P. (2017). Role of Aerosol Liquid Water in Secondary Organic Aerosol Formation from Volatile Organic Compounds. Environ. Sci. Technol..

[19] Kroll J.H., Seinfeld J.H. (2008). Chemistry of secondary organic aerosol: Formation and evolution of low-volatility organics in the atmosphere. Atmos. Environ..

[20] Ziemann P.J., Atkinson R. (2012). Kinetics, products, and mechanisms of secondary organic aerosol formation. Chem. Soc. Rev..

[21] Fine P.M., Sioutas C., Solomon P.A. (2008). Secondary particulate matter in the United States: Insights from the particulate matter supersites program and related studies. J. Air Waste Manag. Assoc..

[22] Whalley L.K., Slater E.J., Woodward-Massey R., Ye C., Lee J.D., Squires F., Hopkins J.R., Dunmore R.E., Shaw M., Hamilton J.F. (2021). Evaluating the sensitivity of radical chemistry and ozone formation to ambient VOCs and NOx in Beijing. Atmos. Chem. Phys..

[23] Berezina E., Moiseenko K., Skorokhod A., Pankratova N.V., Belikov I., Belousov V., Elansky N.F. (2020). Impact of VOCs and NOx on Ozone Formation in Moscow. Atmosphere.

[24] Alvim D.S., Gatti L.V., Corrêa S.M., Chiquetto J.B., Santos G.M., de Souza Rossatti C., Pretto A., Rozante J.R., Figueroa S.N., Pendharkar J. (2018). Determining VOCs Reactivity for Ozone Forming Potential in the Megacity of São Paulo. Aerosol Air Qual. Res..

[25] Lee H., Kim K., Choi Y., Kim D. (2021). Emissions of volatile organic compounds (Vocs) from an open-circuit dry cleaning machine using a petroleum-baseorganic solvent: Implications for impacts on air quality. Atmosphere.

[26] Bidila T., Pietraru R.N., Ionita A.D., Olteanu A. Monitor indoor air quality to assess the risk of COVID-19 transmission. Proceedings of the 2021 23rd International Conference on Control Systems and Computer Science Technologies, CSCS 2021.

[27] Rani R., Arokiasamy P., Sikarwar A. (2021). Household air pollution during COVID-19 pandemic: A concern in India. J. Public Aff..

[28] European Comission (2003). Indoor Air Pollution: New EU Research Reveals Higher Risks than Previously Thought.

[29] Johnson M.M., Williams R., Fan Z., Lin L., Hudgens E., Gallagher J., Vette A., Neas L., Özkaynak H. (2010). Participant-based monitoring of indoor and outdoor nitrogen dioxide, volatile organic compounds, and polycyclic aromatic hydrocarbons among MICA-Air households. Atmos. Environ..

[30] Villanueva F., Tapia A., Lara S., Amo-Salas M. (2018). Indoor and outdoor air concentrations of volatile organic compounds and NO2 in schools of urban, industrial and rural areas in Central-Southern Spain. Sci. Total Environ..

[31] WHO (2012). Burden of Disease from the Joint Effects of Household and Ambient Air Pollution for 2012.

[32] Guo H., Lee S.C., Chan L.Y., Li W.M. (2004). Risk assessment of exposure to volatile organic compounds in different indoor environments. Environ. Res..

[33] (2021). KOSIS. https://kosis.kr/statHtml/statHtml.do?orgId=101&tblId=DT_1KB9001&conn_path=I2.

[34] Kim S., Han J., Kim H. (2001). A Study on the source profile of volatile organic compounds from major emission sources. J. Korean Soc. Atmos. Environ..

[35] Garzón J.P., Huertas J.I., Magaña M., Huertas M.E., Cárdenas B., Watanabe T., Maeda T., Wakamatsu S., Blanco S. (2015). Volatile organic compounds in the atmosphere of Mexico City. Atmos. Environ..

[36] Lam S.H.M., Saunders S.M., Cheng H.R., Guo H. (2015). Examination of regional ozone formation: POCPs for Western Australia and comparisons to other continents. Environ. Model. Softw..

[37] Derwent R.G., Jenkin M.E., Passant N.R., Pilling M.J. (2007). Reactivity-based strategies for photochemical ozone control in Europe. Environ. Sci. Policy.

[38] Derwent R.G., Jenkin M.E., Utembe S.R., Shallcross D.E., Murrells T.P., Passant N.R. (2010). Secondary organic aerosol formation from a large number of reactive man-made organic compounds. Sci. Total Environ..

[39] USEPA (2019). Guideline for Human Exposure Assessment.

[40] Barr D.B., Thomas K., Curwin B., Landsittel D., Raymer J., Lu C., Donnelly K.C., Acquavella J. (2006). Biomonitoring of exposure in farmworker studies. Environ. Health Perspect..

[41] USEPA (2005). Guidelines for Carcinogen Risk Assessment—EPA/630/P-03/001B.

[42] DeLeo P.C., Ciarlo M., Pacelli C., Greggs W., Williams E.S., Scott W.C., Wang Z., Brooks B.W. (2018). Cleaning product ingredient safety: What is the current state of availability of information regarding ingredients in products and their function?. ACS Sustain. Chem. Eng..

[43] Salhotra A.M. (2012). Human health risk assessment for contaminated properties. Prog. Mol. Biol. Transl. Sci..

[44] USEPA (2022). Integrated Risk Information System (IRIS) Glossary. https://sor.epa.gov/sor_internet/registry/termreg/searchandretrieve/glossariesandkeywordlists/search.do?details=&glossaryName=IRIS%20Glossary.

[45] You S.-J., Choi J.-W., Kim Y.-T. (2012). The Health Risk Assessment of Ambient VOCs in Busan Industrial Area.

[46] USEPA (2001). Risk Assessment Guidance for Superfund (RAGS) Volume III—Part A: Process for Conducting Probabilistic Risk Assessment, Appendix B.

[47] Lee T.J., Lee S.M., Chae J.S., Jeon J.M., Kim D.S., Jo Y.M. (2021). Inventory of ozone precursor VOCs from organic solvents used in residential workplaces and assessment of ozone formation contribution. J. Korean Soc. Atmos. Environ..

[48] Vega E., Mugica V., Carmona R., Valencia E. (2000). Hydrocarbon source apportionment in Mexico City using the chemical mass balance receptor model. Atmos. Environ..

[49] Scheff P.A., Wadden R.A., Bates B.A., Aronian P.F. (1989). Source fingerprints for receptor modeling of volatile organics. J. Air Pollut. Control Assoc..

[50] Achimón F., Brito V.D., Pizzolitto R.P., Zygadlo J.A. (2022). Effect of carbon sources on the production of volatile organic compounds by fusarium verticillioides. J. Fungi.

[51] Wang J.Z., Hou Y., Zhang J., Zhu J., Feng Y.L. (2013). Transformation of 2,2’,4,4’-tetrabromodiphenyl ether under UV irradiation: Potential sources of the secondary pollutants. J. Hazard. Mater..

[52] Palmisani J., Nørgaard A.W., Kofoed-Sørensen V., Clausen P.A., de Gennaro G., Wolkoff P. (2020). Formation of ozone-initiated VOCs and secondary organic aerosol following application of a carpet deodorizer. Atmos. Environ..

[53] Omrane F., Khadhraoui M., Abid A., Mitigui M., Elleuch B., Gargouri I. (2021). Health risk assessment of occupational exposure to perchloroethylene and trichloroethylene in dry cleaning in Sfax city (Tunisia). Environmental Science and Engineering.

