# Supplementary material for: Analysis of VOCs Emitted from Small Laundry Facilities: Contributions to Ozone and Secondary Aerosol Formation and Human Risk Assessment
Source: Int J Environ Res Public Health. 2022 Nov 16;19(22):15130. doi: 10.3390/ijerph192215130 (PMC9691109; doi:10.3390/ijerph192215130)
Supplement: Supplementary file 1 [file ijerph-19-15130-s001.zip › ijerph-1997447-supplementary.pdf]

Supplementary Information

Analysis of VOCs Emitted from Small Laundry Facilities: Contributions to Ozone and Secondary Aerosol Formation and Human Risk Assessment

Da-Mee Eun [1], Yun-Sung Han [1], Soo-Hyun Park [1], Hwa-Seong Yoo [2], Yen Thi-Hoang Le [3,4], Sangmin Jeong [5], Ki-Joon Jeon [3,4,6,*] and Jong-Sang Youn [1,*]

[1] Department of Energy and Environmental Engineering, The Catholic University of Korea,
Bucheon 14662, Republic of Korea
[2] Lab.SolEmis, Incheon 22212, Republic of Korea
[3] Department of Environmental Engineering, Inha University, Incheon 22212, Republic of Korea
[4] Program on Environmental and Polymer Engineering, Inha University, Incheon 22212, Republic of Korea
[5] Department of Chemistry, University of Massachusetts, Lowell, MA 01854, USA
[6] Particle Pollution Research and Management Center, Incheon 21999, Republic of Korea
* Correspondence: kjjeon@inha.ac.kr (K.-J.J.); jsyoun@catholic.ac.kr (J.-S.Y.)

Table S1. Characteristics of adsorbent used for VOCs sampling.

Sorbents	Range of adsortion	Max. temp. (°C)	Strength	Pretreatment condition
Carbopack C	n-C8 to n-C20	> 400	Weak	350°C, 2hr
Carbopack B	(n-C4) n-C5 to n-C12	> 400	Medium	350°C, 2hr
Carbosieve SIII	n-C2 to n-C5	400	Strong	350°C, 2hr

Table S2. Chemical characteristics and method detection limit (MDL) of the analytes.

No.	Compound	M.W	B.P (°C)	R.T (min)	MDL (ng)
1	Chloromethane	50.49	-24.2	3.396	1.16
2	Freon 114	170.93	4.1	3.478	0.67
3	Vinyl chloride	62.5	-13.4	3.668	2.15
4	1,3-Butadiene	54.0916	-4.4	3.828	3.98
5	Bromomethane	94.94	3.6	4.217	2.45
6	Chloroethane	64.52	12.3	4.42	2.29
7	Freon 11	137.38	23.7	5.416	3.87
8	Acrylonitrile	53.06	77	5.716	2.42
9	1,1-Dichloroethene	96.95	31.7	6.327	4.77
10	Methylene chloride	84.94	39.8	6.47	0.88
11	3-Chloropropene	76.53	45.1	6.649	6.06
12	Freon 113	187.38	47.7	6.858	1.39
13	1,1-Dichlorethane	98.96	57.3	8.406	7.29
14	Cis-1,2-Dichloroethylene	96.94	60.3	10.141	2.65
15	Chloroform	119.38	61.7	10.796	5.30
16	1,2-Dichloroethane	98.96	83.5	12.493	3.13
17	Benzene	78.12	80.1	14.156	0.54
18	Carbon Tetrachloride	153.82	76.5	14.506	3.29
19	1,2-Dichloropropane	112.99	96.4	15.984	3.02
20	Trichloroethylene	131.29	87	16.529	1.71
21	Cis-1,3-Dichloropropene	110.97	104.3	18.446	7.64
22	Trans-1,3-Dichloropropene	110.97	112	19.629	5.29
23	1,1,2-Trichloroethane	133.41	113.8	20.043	0.76
24	Toluene	92.15	110.6	20.725	0.60
25	1,2-Dibromoethane	187.88	131.3	22.27	4.43
26	Chlorobenzene	112.56	132	24.956	0.34
27	Ethylbenzene	106.17	136.2	25.851	0.50
28	m&p-Xylene	106.17	138.3	26.293	0.39
29	Styrene	104.16	145.2	27.162	0.25
30	1,1,2,2-Tetrachloroethane	106.17	144.4	27.428	5.63
31	o-Xylene	167.85	146.2	27.393	0.31
32	4-Ethyltoluene	120.19	162	30.662	0.41
33	1,3,5-Trimethylbenzene	120.2	164.7	30.882	0.59
34	1,2,4-Trimethylbenzene	120.2	169.3	32.027	0.54
35	1,3-Dichlorobenzene	147.01	173	32.484	0.54
36	1,4-Dichlorobenzene	147.01	174	32.679	0.88

37	1,2-Dichlorobenzene	147.01	180.5	33.701	0.56
38	1,2,4-Trichlorobenzene	181.45	213.5	39.322	2.40
39	Hexachloro-1,3-Butadiene	260.8	186	40.943	5.63
40	tert-Butyl methyl ether	88.15	55.2	8.468	6.28
41	Dibromomethane	173.83	96.95	15.951	1.58
42	2-Hexanone	100.161	128	21.29	8.06
43	Ethane, 1,1,1,2-tetrachloro-	167.848	146.5	24.957	3.79
44	Bromoform	252.73	149.1	26.532	1.77
45	Propane, 1,2,3-trichloro-	147.43	156	27.775	0.00
46	Nitrobenzene	123.11	210.9	35.275	0.31
47	Naphthalene	128.1705	218	39.771	1.53
48	Ethanol	46.07	78.37	4.551	2.86
49	2-Propanol	60.1	82.5	5.428	2.00
50	1-Propanol	60.0952	97	7.511	1.72
51	2-Butanone	72.11	79.64	9.098	2.50
52	Ethyl Acetate	88.11	77.1	10.515	1.23
53	Hexane	86.18	69	10.617	2.84
54	2,4-Dimethylpentane	100.2	80.4	12.661	0.59
55	1-Butanol	74.12	117.7	13.839	1.26
56	Bromodichloromethane	163.8	90	16.44	0.69
57	2,2,4-Trimethylpentane	114.23	99	16.657	0.57
58	Heptane	100.21	98.42	17.215	0.55
59	4-Methyl-2-pentanone	100.16	116	18.523	0.97
60	Dibromochloromethane	208.28	120	21.713	0.60
61	Octane	114.23	125.6	23.022	0.60
62	Nonane	128.2	151	27.984	1.19
63	(−)-α-Pinene	136.24	155	29.992	2.80
64	3-Ethyltoluene	120.19	161.1	30.581	0.82
65	2-Ethyltoluene	120.2	164	31.441	0.82
66	(−)-β-Pinene	136.23	165	31.878	0.49
67	Decane	142.29	174.1	32.329	1.44
68	1,2,3-Trimethylbenzene	120.19	176	33.326	0.92
69	(*R*)-(+)-Limonene	136.23	176	33.752	1.87
70	Nonanal	142.2386	191	35.761	3.24
71	Undecane	156.31	196	36.235	1.45
72	Durene	134.22	192	37.033	0.95
73	Decanal	156.2	207	39.463	2.16
74	Dodecane	170.33	216.2	39.775	0.80
75	Tridecane	184.37	234	43.059	0.80
76	Tetradecane	198.39	253.6	46.297	0.93
77	Pentadecane	212.42	270.6	50.121	1.76

M.W : Molecular weight, B.P : Boiling point, R.T : Retention time, MDL : Method detection limit

Table S3. Information and health impact of toxic carcinogen VOCs.

No.	Compound	tumor type	Test species	Route	Reference	Last revised
1	Acrylonitrile	Respiratory cancer	humans	inhalation	O'Berg, 1980	09/30/1987
2	Methylene chloride	Hepatocellular carcinomas or adenomas, bronchoalveolar carcinomas or adenomas	Male B6C3F1 mice	Inhalation	Mennear et al., 1988; NTP, 1986	11/18/2011
3	Benzene	Leukemia	Humans	Inhalation	Rinsky et al., 1981, 1987; Paustenbach et al., 1993; Crump and Allen, 1984; Crump, 1992, 1994; U.S. EPA, 1998.	01/19/2000
4	Carbon Tetrachloride	pheochromocytoma	male BDF1 mouse	inhalation	Nagano et al. 2007b; JBRC 1998	03/31/2010
6	Nitrobenzene	liver hepatocellular adenomas or carcinomas, kidney tubular adenomas or carcinomas, thyroid follicular cell adenomas or carcinomas	rat/F344, male	inhalation	CIIT, 1993	02/06/2009
7	Bromodichloromethane	kidney (tubular cell adenoma and tubular cell adenocarcinoma)	B6C3F1 mice, male	oral	IRIS, 3/01/1993 and PPRTV, 9/16/2009.	11/18/2015
8	4-Methyl-2-pentanone	mononuclear cell leukemia	male rat	inhalation	Henning et al, 2014	10/17/2017
9	Chloromethane	renal cortical adenomas and adenocarcinomas	male B6C3F1 mice	inhalation	CCD/WRD, 1/11/2000	09/16/2015

Table S4. Information and health impact of toxic non-carcinogen VOCs.

No.	Compound	Influence system	Critical effect	Test species	Route	Last revised
1	Bromomethane	Nervous, Respiratory	Degenerative and proliferative lesions of the olfactory epithelium of the nasal cavity	Rat 29-month Inhalation Study	Inhalation	04/01/1992
2	Toluene	Nervous	Neurological effects in occupationally-exposed workers	Humans	Inhalation	09/23/2005
3	Chlorobenzene	Respiratory	respiratory tract irritation, drowsiness and dizziness	Humans	Inhalation	02/18/2008
4	m&p-Xylene	Nervous	Impaired motor coordination (decreased rotarod performance)	Subchronic inhalation study in male rats	Inhalation	02/21/2003
5	o-Xylene	Nervous	Impaired motor coordination (decreased rotarod performance)	Subchronic inhalation study in male rats	Inhalation	02/21/2003
6	1,3-Dichlorobenzene	Respiratory, Hepatic	respiratory tract, liver damage, headaches and dizziness	Humans	Inhalation	2006
7	1,2,4-Trichlorobenzene	Respiratory	respiratory tract	Rat and rabbit	Inhalation	11/2003
8	Hexachloro-1,3-Butadiene	Respiratory, Urinary	Renal toxicity, respiratory tract	Rat	Inhalation	03/2021
9	Ethanol	Nervous, Respiratory	respiratory tract, dizziness, nausea, headache	Humans	Inhalation	09/26/2007
10	Hexane	Nervous	Peripheral neuropathy (decreased MCV at 12 weeks)	Rat subchronic inhalation study	Inhalation	12/23/2005
11	Ethylbenzene	Developmental	Developmental toxicity	Rat and rabbit developmental inhalation studies	Inhalation	03/01/1991

Table S5. Concentration of VOCs emitted from one cycle of dry cleaning process.

(unit : ppb)

No.	Compound	Conc.±S.D.	No.	Compound	Conc.±S.D.
1	Nonane	409.19±36.17	25	(−)-β-Pinene	1.38±0.17
2	Decane	319.94±17.94	26	2,4-Dimethylpentane	1.38±1.95
3	Undecane	127.35±5.42	27	Hexachloro-1,3-Butadiene	1.35±1.91
4	Nonanal	54.15±5.49	28	tert-Butyl methyl ether	1.28±0.03
5	Decanal	29.13±1.82	29	(−)-α-Pinene	1.05±0.13
6	o-Xylene	23.76±11.06	30	1,1,2,2-Tetrachloroethane	1.02±0.26
7	Chlorobenzene	23.58±6.42	31	Ethylbenzene	1.01±0.15
8	(R)-(+)-Limonene	19.3±2.47	32	1,2,4-Trimethylbenzene	0.99±0.05
9	Ethanol	12.45±3.44	33	2-Butanone	0.92±0.06
10	Nitrobenzene	10.73±0.77	34	m&p-Xylene	0.87±0.1
11	Bromomethane	8.18±5.79	35	1,2,4-Trichlorobenzene	0.73±0.52
12	Dodecane	7.12±0.48	36	Bromodichloromethane	0.61±0.86
13	Hexane	6.02±0.24	37	1,3,5-Trimethylbenzene	0.56±0.03
14	Chloromethane	5.78±2.35	38	Styrene	0.53±0.03
15	Octane	4.22±0.73	39	Benzene	0.47±0.12
16	Acrylonitrile	3.8±0.11	40	Vinyl chloride	0.46±0.65
17	1-Butanol	3.68±0.2	41	Tetradecane	0.25±0.05
18	2,2,4-Trimethylpentane	3.62±0.38	42	1,3-Dichlorobenzene	0.21±0.02
19	Ethyl Acetate	3.44±0.05	43	Pentadecane	0.17±0.12
20	Heptane	3.32±0.05	44	Chloroform	0.17±0.24
21	Toluene	2.62±0.21	45	Freon 113	0.04±0.06
22	Methylene chloride	2.08±0.27	46	1,2-Dichlorobenzene	0.02±0.02
23	4-Methyl-2-pentanone	1.82±0.04	47	2-Propanol	0.01±0.01
24	Carbon Tetrachloride	1.77±0.01			
